# Improved Cholinergic Transmission is Detrimental to Behavioural Plasticity in Honeybees (*Apis mellifera*)

**DOI:** 10.3390/biology1030508

**Published:** 2012-10-16

**Authors:** David Guez, Hong Zhu, Shao-Wu Zhang

**Affiliations:** 1 School of Psychology, The University of Newcastle, Callaghan, NSW 2308, Australia; 2 Research School of Biology, The Australian National University, Canberra, ACT 0200, Australia; Email: Hong.hongzhu.zhu@gmail.com (H.Z.); Shaowu.zhang@anu.edu.au (S.-W.Z.)

**Keywords:** pesticide, organo-phosphate, nicotinic, muscarinic, recall, learning reversal, *Apis mellifera ligustica*

## Abstract

Unravelling the role of neuromessenger processes in learning and memory has long interested researchers. We investigated the effects of an acetylcholinesterase blocker, Methyl Parathion (MeP), on honeybee learning. We used visual and olfactory tasks to test whether MeP had a detrimental effect on the acquisition of new knowledge when this new knowledge contradicts previously acquired one. Our results indicate that treatment with MeP prior to conditioning was significantly detrimental to the acquisition of incongruous (but not irrelevant or congruous) new knowledge due to improved recall. The neurobiological and ecotoxicological consequences of these results are discussed.

## 1. Introduction

Understanding how different pharmacological compounds impact upon neuromessenger system processes and affect an organism’s learning and memory has long been an interest of animal behaviour and cognitive science researchers. It is now known that many chemical factors including neurotransmitters influence an organisms learning and memory through their action on different regions of the brain. For example, in mammalian cholinergic systems nicotinic pathways have been found to be involved in learning and memory [1]. Nicotinic antagonists have been found to impede memory function [2], whilst nicotinic agonists have been found to improve performance on a variety of learning and memory tasks such as enhancing acquisition of fear conditioning, eye-blink conditioning or the acquisition of water maze spatial navigation [3,4], medium term retention [3] and working memory performance [2]. Muscarinic antagonist treatments such as scopolamine have been found to be detrimental to recall [2], whilst muscarinic agonist treatments have been found to facilitate both working memory and cognitive flexibility [5].

The involvement of the cholinergic and aminergic systems in learning and memory has also been extensively investigated using invertebrate models, such as the honeybee (*Apis mellifera*) [6,7,8,9,10,11,12,13,14,15]. The majority of these studies have utilised the olfactory conditioning of the proboscis extension reflex (PER) [16] or its habituation. For example, Braun *et al.* [17] studied the effect of eserine, an acetylcholinesterase blocker, on habituation and showed that eserine treatment significantly altered the performance of bees in the habituation of PER by increasing the number of trials before the onset of habituation. More recently, Behrends and Scheiner [18] used PER to study the effect of octopamine on newly emerged bees and found that it greatly improved learning performance.

Honeybees have also been used to further explore the link between the nicotinic and muscarinic systems and learning and memory. It has been shown that nicotinic agonists such as Imidacloprid (a neonicotinoid) significantly impact upon the habituation of the PER in young non-foraging bees and that results can be age dependent [13,14]. In 7 day-old bees, it was found that treatment with Imidacloprid resulted in an increase in the number of trials before habituation. In comparison, with 8 day-old bees the same treatments led to a decrease in the number of trials before habituation 15 minutes and 1 hour after treatment, and an increase in the number of trials 4 hours after treatment. However for older, foraging bees, Imidacloprid treatments led to a decrease in the number of trials before habituation 1 hour after treatment [6]. Other studies have also found that treatment with nicotinic antagonists compromised acquisition as well as recall [8,9,10] suggesting that the nicotinic system plays a large role in the acquisition and retrieval of knowledge. However, treatments with muscarinic antagonists were found to only compromise the recall process, suggesting that the muscarinic system is involved solely in this learning process [8,9,10].

In agriculture, insecticide families of broad commercial interest such as organo-phosphates, carbamates, and neonicotinoids target an insect’s cholinergic system [19]. Organo-phosphates such as Methyl Parathion (MeP) and carbamate irreversibly or reversibly block their acetylcholinesterase, resulting in cholinergic up regulation [19]. Neonicotinoids are super or partial agonists of the nicotinic receptors to acetylcholine [20,21,22]. Therefore, increased levels of cholinergic transmission mediate the lethal effects of these families of pesticides. In agriculture honeybees are a non-target species that are crucially important for the pollination of certain crops. A better knowledge of the behavioural consequences of non-lethal doses of cholinergic agents is therefore of prime importance for ecotoxicology and for the development of better methods for pesticide use. 

**Figure 1:**
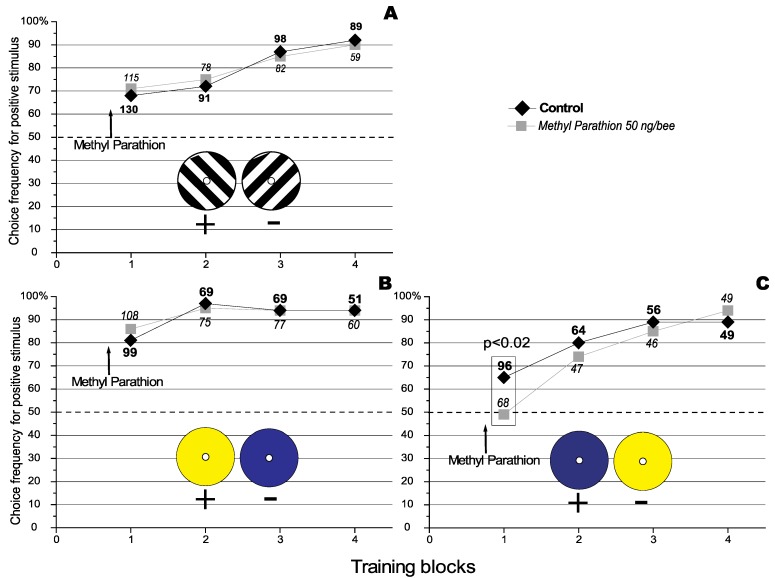
Effect of Methyl Parathion on the acquisition of a visual orientation discrimination task. The number against each data point represents the total number of choices analysed for that particular condition. The number of choices that were analysed under the different conditions are represented using different fonts types (bold for the control, and in italics for the treatment).The dashed line represents the level of random choice (50%). (**A**) where the 135° black and white grating is the positive stimulus (Redraw from [15] with permissions of the *Journal of Insect Physiology*). The results represent data pooled from 3 experiments using a total of 48 individually marked bees. (**B**) where the yellow target is the positive stimulus. The results represent data pooled from 3 experiments using a total of 62 individually marked bees. (**C**) where the blue target is the positive stimulus. The results represent data pooled from 3 experiments using a total of 38 individually marked bees.

In a previous paper it was observed that Methyl Parathion (MeP), an anti acetylchlolinesterase agent, specifically improved the recall of previously learnt discriminations in the honeybee (*Apis mellifera*) in both a visual and an olfactory discrimination task without affecting acquisition or consolidation [15]. It was also observed that MeP had no effect on the acquisition of a visual discrimination task using differently orientated stimuli (see Figure 1, Panel A). However here we report recent conflicting results. Finally we propose and successfully test a possible explanation for the apparent discrepancy. 

## 2. Results

### 2.1. Visual Discrimination Learning

In earlier work it was shown that Methyl parathion (MeP) did not affect the acquisition of new knowledge but instead affected the recall of a previously acquired rule [15]. Figure 1 shows the effect of topical application of MeP (50 ng/bee) 10 minutes before training in a visual discrimination task. Panel A, Figure 1 shows that treatment with MeP had no effect on the acquisition of a visual grating discrimination, and Panel B, Figure 1 shows that the same treatment had no effect on the acquisition of a colour discrimination when the yellow target was reinforced. Surprisingly, when the blue target was reinforced, treatment with MeP was found to have a detrimental effect on the acquisition of this simple discrimination (Figure 1, Panel C). The question here is: why should the choice of the reinforced target change how a honeybee reacts to a MeP challenge? 

If we observe the results presented in Figure 1 we can note that the control groups for the grating experiment (no colour, Panel A) and when the blue target was reinforced (Panel C) learned in similar manner. After the first learning block the controls for both of these groups chose the positive target 70% of the time. In contrast, when the yellow target was reinforced (Panel B), the control group chooses the positive target 80% of the time after the first block. This suggests that although the discrimination tasks presented in Panels A and C appears to be of similar difficulty, the discrimination involving reinforcement of the yellow target was actually easier for the honeybees. Interpreting these differences is difficult since the experiments were not run simultaneously. Nevertheless, as the data presented was pooled across at least 3 repetitions, any variation in environmental factors should be greatly diminished. However, it is not possible to exclude a possible contribution of previously acquired knowledge on the performance displayed by the honeybee in this visual discrimination task.

### 2.2. Olfactory Discriminations Learning

Honeybees visiting our maze in our visual discrimination task are foragers and therefore have previous floral experience that we are unable to control. Therefore they may have had previous exposure to yellow flowers. It is possible therefore that MeP improved recall and induced bees to persist in their acquire original preference for yellow over blue. Therefore, treated bees would appear to display a failure to acquire the discrimination when blue is the positive target and yellow the negative target (as seen in Figure 1, Panel C). This idea of an acquired preference for yellow is further supported by the fact that it seems easier for bees to discriminate in favour of the yellow target when it was the positive stimulus in the discrimination (Figure 1, Panel B). This assertion is difficult to test because it is impossible to know the exact foraging history of the bees used in the test, particularly in terms of what proportion of flower colour they have previously visited. However, it is possible to determine this in the olfactory domain, as was done in the present part of our experiment

**Figure 2:**
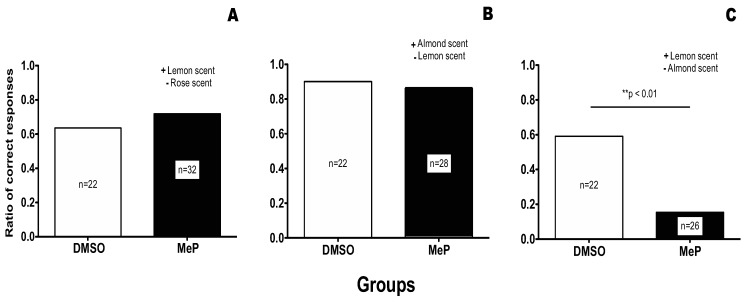
Effect of Methyl Parathion treatment on an olfactory discrimination task. (**A**) the acquisition of a discrimination task that is neutral to previous knowledge (Irrelevant condition). (**B**) the acquisition of a discrimination task that is consistent with previous knowledge (Consistent condition). (**C**) the acquisition of a discrimination task that contradicts previous knowledge (Inconsistent condition).

To ensure that bees would have prior knowledge of the association of a smell (here an almond scent) and a sugar reward, we provided forager bees with a feeder scented with almond essence. We marked forager bees that visited the feeders three days prior to the experiment and then collected the marked bees for use in conditioning on the morning of the experiment (see methods for full details). This method ensured that the collected forager honeybees would have had multiple conditioning trials (>300 trials) associating an almond scent with a sugar reward. (Interestingly, often a single odour associative trial in olfactory conditioning is known to result in up to 80% conditioning on its own.) Collected bees were then subjected either to (i) an irrelevant discrimination conditioning training where the almond scent was not involved in the discrimination (Figure 2, Panel A), (ii) a consistent discrimination training where the almond scent was the positive stimulus; Figure 2, Panel B), or to (iii) an inconsistent discrimination training where the almond scent was associated with the negative stimulus.

In the Irrelevant condition (Figure 2, Panel A), prior association of the almond scent with a sugar reward could not interfere with the new discrimination. For this test the positive stimulus was lemon scent and the negative stimulus was rose scent. As shown in Figure 2 Panel A, no significant difference was found between the DMSO control group and the treatment group, leading us to the conclusion that treatment with MeP does not affect the acquisition of new knowledge in the irrelevant condition.

In the consistent condition (Figure 2, Panel B), prior association of the almond scent with a sugar reward could only facilitate the acquisition of the discrimination since almond was again associated with a sugar reward. In fact, the only association left for the bees to make was that the lemon scent was associated with the saturated salt solution. As shown in Figure 2 Panel B, both the DMSO control and the treated group acquired this discrimination. Also, this discrimination was much easier for the bees to acquire than the totally new discrimination of the irrelevant condition as was expected. As can be seen in Figure 2, the control group in the consistent condition achieved 90% of correct responses, whereas in the Irrelevant condition the control group achieved only 63.64% of correct responses.

The experimental results obtained when the olfactory discrimination training contradicted previous knowledge (*i.e.*, the previous association of an almond scent with a sugar reward), is shown in Figure 2, Panel C. In this case, treatment with MeP prior to conditioning was significantly detrimental to the acquisition of the discrimination (compare the DMSO group with the treatment group in Figure 2 Panel C, Fisher exact test *p* < 0.01).

## 3. Material and Methods

### 3.1. Y-Maze Experiments

**Figure 3:**
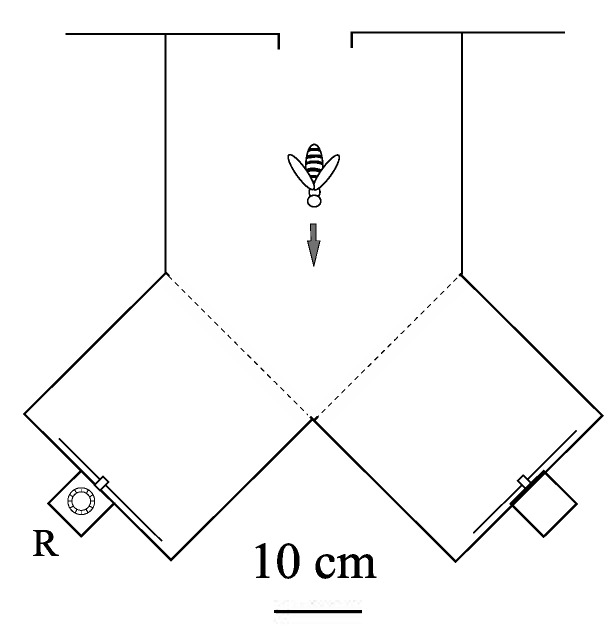
Plan view of Y-maze (transparent plexiglass top, sides and bottom). Each stimulus was presented in the vertical plane on the end wall of one arm. The feeder was placed in the reward box, R, behind the positive stimulus. A similar box, carrying no reward, was placed behind the negative stimulus. The visual angle offered by each stimuli was 25°.

All experiments were performed in the all weather bee flight facility at the Australian National University, Canberra. Forager bees (*Apis mellifera ligustica*) were first trained to enter a Y-Maze and visit a sugar feeder placed in the maze. The feeder was progressively placed further into the maze and its placement was alternated in both branches of the maze. The feeder was then placed in the reward box at the back of the apparatus (see Figure 3), and the bees were trained to enter the reward box in order to gain access to the feeder. The feeder position alternated between each reward box (grey targets were present at the front of each reward box and therefore could not be used to discriminate between the non-reward and the reward box). Getting the bees to enter the reward box was the longest part of the pretraining. Bees who accessed the feeder at this stage were individually marked using non-toxic paint, and were trained to discriminate between two visual stimuli, each presented in one arm of a Y‑Maze (Figure 3). The stimuli differed either in orientation (as in Figure 1, Panel A) or in colour (as in Figure 1, Panel B and C). In each experiment, one stimulus (termed the positive stimulus, +) was associated with a reward of sugar solution provided by a feeder placed in a box behind the stimulus. The other stimulus (termed the negative stimulus, −) carried no reward. Bees could access the reward by entering the box via a tube passing through the centre of the stimulus. During training and testing the positions of the two stimuli were regularly interchanged. This ensured that the bees learned to associate the reward with the positive stimulus, and not with a particular arm of the Y-maze. 

Although our experimental beehive was kept indoors within temperature controlled facilities, the bees were given access to the outside environment through a small exit tube. Therefore, the activity of the hive was still greatly influenced by outside weather conditions. Because of this we were unable to train the bees individually as we could not ensure that the weather conditions for both the control and treatment sets would be exactly the same. Therefore, both control and treatment sets were trained simultaneously. For this reason, a training block was deemed to be complete when each stimulus had occupied each arm of the Y-maze once and not after a specified number of trials for each individual bee. The total duration of the first training block was 30 minutes, with each stimulus remaining in a given arm for 15 minutes (with an average of 3 visits per bee per 15 minutes). The duration of each subsequent training block was 20 minutes, with each stimulus remaining in a given arm for 10 minutes (with an average of 2 visits per bee per 10 minutes). The choice performance of each marked bee was continuously monitored from the commencement of the training. The performance was scored by recording the first choice of each bee after it entered the Y-maze. If a bee entered the arm containing the positive stimulus, it was regarded as a correct choice (such a visit invariably resulted in the entry of the reward box and reinforcement). An entry into the arm containing the negative stimulus was regarded as an incorrect choice. The choice frequency in favour of the positive stimulus was calculated separately for each block as 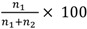, where n_1_ and n_2_ denote the number of correct and incorrect choices, respectively. Control tests, carried out by temporarily removing the feeder from its usual location behind the positive stimulus, assured us that the bees were not choosing the correct stimulus on the basis of pheromone scents deposited on the feeder. Further details of training and testing procedures for Y-mazes are given in [23].

Each training/testing experiment was carried out at least three times using a fresh set of bees each time. The figures show the choice frequencies in favour of the positive stimulus for each training block, obtained by pooling choices across bees and across repeated experiments (since the same trend was observed across all repetitions). 

#### 3.1.1. Treatment

Bees were treated by topical application of 1 μL of MeP (Sigma) solution dissolved in DMSO ([MeP] = 50 mg/L). The DMSO (solvent) functioned as a transport medium for the drug by facilitating the transfer of the drug through the insect cuticle. The drug was applied on the dorsal side of the thorax while the bees visited and drank at the feeder during a ten minutes period prior to acquisition (Figure 1). Since preliminary experiments and previously published works showed that DMSO did not elicit any observable behavioural effects in free flying conditions [24], and given the experimental constraints that precluded the simultaneous use of a large number of bees in order to follow them accurately, controls with DMSO were not included. Pure DMSO has also been shown to have no effect on other aspects of honeybee behaviour, such as habituation [13,14]. The only claim of an effect of DMSO on honeybee behaviour can be found in [6] on habituation, although this effect was obtained only when DMSO was mixed with a saline solution and not on its own (see also [13,14]).

Our choice of using topical application over injection was based upon two factors. Firstly, we were using honeybees in free flying conditions, which made the use of injections unpractical. As noted by Barron *et al.* [25], topical application is technically easier and much less stressful than injection for the animal, as it does not involve anaesthetic treatments. Secondly, injection and anaesthesia treatment in insects are known to cause an immune response and/or behavioural changes [26,27,28]. Mallon *et al.* [27] have also showed that an induced immune response in honeybee inhibited associative learning. Because we were studying the effect of MeP on associative learning in honeybees, treatment by injection was therefore ruled out and topical application was preferred (this argument was also valid for our olfactory conditioning experiments).

The dose of MeP used was chosen based on the results obtained in a previous experiment [15,24]. In this previous work complex time dependent effects were observed for the lowest dose of MeP, namely 10 ng/bee (29 time less than the LD50), compared to the highest dose of 50 ng/bee (6 time less than the LD50). The time variation of effects observed at the two doses was attributed to the fact that the critical concentration at the site of action was reached at different times after the application of the pesticide. To minimise this factor in the present experiments the 50 ng/bee dose was chosen. No mortality was observed at these doses for the duration of any of these experiments (present and past). 

#### 3.1.2. Statistical Analysis

χ^2^ tests were performed using the Systat [29], to check for significant differences in choice frequency between the treated and control groups. This was done separately for each training block. *p* values lower than 0.05 were considered as significant. Data were pooled across all repetitions since response trends were found to be consistent across all repetitions.

### 3.2. Olfactory Conditioning Using the Proboscis Extension Reflex

Forager bees (*Apis mellifera ligustica*) were marked three days before collection on an almond scented feeder. On the day of the experiment the marked bees were collected on the same feeder. The bees were immobilised on ice immediately after capture and mounted in thin-walled aluminium tubes (7 mm inner diameter) using a thin strip of fabric-reinforced tape (GAFFA) with the thorax exposed. After mounting, the bees were fed to satiation with 1 M sugar solution, placed along rows in a Perspex holder and kept for four hours in an incubator (31 °C).

Bees were trained to distinguish between a positive (reward-bearing, sucrose solution) odour and a negative (non reward-bearing, saturated NaCl solution) odour. The training procedure was as in [30], but performed in one trial (see below). The training procedure was therefore a modified version of the well-known olfactory conditioning protocol of the PER, which usually involves training to a single odour [16,31] and the 3 trials procedure proposed by Maleszka with a ‘positive’ and a ‘negative’ odour [30]. This 1 trial discrimination method is used to ensure that the response at test is the result of learning and not simply due to an increased spontaneous response to odour stimulation.

In all our experiments the positive odour was a natural essence (4 μL/mL), dissolved in 1 M sucrose solution, which constituted the reward. The negative odour was a second natural essence (4 μL/mL), dissolved in saturated NaCl solution, which constituted the non-reward solution [30]. In the neutral prior knowledge group the ‘positive odour’ and ‘negative odour’ were lemon (+) and rose (−) essence, respectively. In the consistent prior knowledge group the scents were almond (+) and lemon (−) respectively and in the Inconsistent prior knowledge group they were lemon (+) and almond (−) respectively.

Testing was always carried out 1 hour after the training to ensure that it was well after the consolidation of the memory trace. A test consisted of waving a drop of the positive odour solution and a drop of the negative odour solution in front of the antennae. Each drop was then presented, in turn, for 5 seconds. The order of the presentation (positive odour followed by negative odour, or *vice versa*) was randomised for each test and each bee was tested only once. The test stimuli carried no sugar reward or salt detriment, but possessed the same odour concentration as the training stimuli. At the end of each test (after presentation of both odours), the persistence of the reflex was checked by touching the bee antennae with a 1 M sucrose solution. Data from bees that failed to respond to a 1 M sucrose solution antennal stimulation with a proboscis extension were disregarded. We had expected that a failure to exhibit the PER at this stage may have been the expression of a physiological fatigue that precluded the expression of learning, but this case did not present itself during the course of the experiment.

The test results were scored as follows. (i) If the positive odour elicited a proboscis extension but the negative did not, the test trial was regarded as having yielded a correct response. (ii) If both odours elicited a proboscis extension, the response was regarded as incorrect. (iii) If the negative odour elicited a proboscis extension but there was none with the positive odour, the response was regarded as incorrect (this response rarely occurred). (iv) If neither odour elicited a proboscis extension, the response was discounted. (Although this last case could be indicative of a memory/learning problem, the absence of a proboscis extension to either odour could also have been due to lack of olfactory sensitivity. Because the two possible cases could not be disambiguated it would have been inappropriate to label either of these as correct or incorrect responses and so they were omitted. Such a case did not present itself in the work reported herein. The frequency of correct responses in the tests was calculated as 
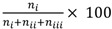
, where n_i_, n_ii_ and n_iii_, and denote the number of responses in categories (i), (ii) and (iii), respectively. 

Each training/testing experiment was carried out at least three times, using a fresh set of bees each time. Training and testing were carried out with the experimental bee placed in front of a suction fan to extract the odours and thus prevent the air in the experimental area from becoming saturated with them. 

#### 3.2.1. Treatment

We used MeP (Sigma) in DMSO solution. After mounting, bees were treated by topical application of 1 μL of DMSO alone or MeP in DMSO on the dorsal side of the thorax for a total dose of MeP of 50 ng/bee. The control bees were treatment free. The treatment was applied 10 minutes before the learning trial (acquisition). The bees were tested for odour discrimination 1 hour after training.

#### 3.2.2. Statistical Analysis

Fisher exact tests tests were performed using Prism 5 [32], to check for significant differences in choice frequency between the treated and control groups. *p* values lower than 0.05 were considered as significant.

## 4. Discussion and Conclusion

This work provides important insights into the brain processes that underlie the representational processes used to guide behaviour. Particularly, these results illustrate that the way animals recall past experiences can greatly influence the observed performance of new learning tasks. This problem may be particularly acute when observing the effects of pharmacological agents on learning. For example, if the effect of MeP treatment on the acquisition of a visual blue-yellow discrimination test as described here was taken at face value, it would suggest that MeP had a detrimental effect on acquisition. However, as it has been shown, this effect was instead an artifact of an improvement in the recall of past learning experiences. Perhaps counter intuitively, our results suggest that an improvement of recall may result in a decrease of behavioural flexibility—a phenomenon that could have many implications in natural populations.

At a neurobiological level, these results are intriguing, as it would be expected that treatment with MeP should increase both nicotinic and muscarinic system activity by increasing the general availability of the endogenous ligand acetylcholine. Cano Lozano *et al.* and Gauthier *et al.* [8,9,10] have shown that treatment with antagonists of the nicotinic system are both detrimental to both acquisition and recall, whereas treatment with antagonists of the muscarinic system are only detrimental to recall. Furthermore, studies that used a partial agonist of the nicotinic system (Imidacloprid) showed that agonistic stimulation were detrimental to the acquisition process [33]. However, treatment with MeP only affected the recall process to the exclusion of the acquisition. Therefore, it would appear that treatment with MeP only mimics the effect of treatment with a muscarinic agonist [34] to the exclusion of the detrimental effect that is observed following treatment with both an agonist and antagonist of the nicotinic system. 

Guez *et al.* [15] put forward a hypothesis that was able to explain this discrepancy based on a muscarinic autoreceptor that down regulates the nicotinic pathway. If such was the case it is expected that treatment with an acetylcholinesterase blocker would only mimic the expected agonistic effect on the muscarinic pathway, while the agonistic effect on the nicotinic pathway would be masked by its down-regulation by the muscarinic system at the presynaptic level. If this hypothesis were true one would expect that concomitant treatment using an inhibitor of acetylcholinesterase (an antagonist of the muscarinic receptor in honeybees) should allow the expression of a nicotinic effect because of the increase availability of acetylcholine in the synaptic cleft. Such studies would be facilitated by the fact that genomic analysis of the honeybee genome has revealed the presence of only one muscarinic receptor in the species [35]. 

From an ecotoxicological point of view, the results obtained here together with those presented previously [15] may have implication beyond the behavioural consequences of MeP exposure. Although MeP (an organo-phosphate) is now banned in a lot of countries, insecticide such as carbamates (e.g., Aldicarb, Furadan and Carbaryl) and other organo-phosphates (e.g., Coumaphos, Malathion) are also acetylcholinesterase inhibitors [19]. Thus, it is possible to consider that the exposure of honeybees to these pesticides may result in essentially the same sub-lethal effects on recall and reversal learning deficits that were observed with the use of MeP.

If other acetylcholinesterase inhibiting, organo-phosphate or carbamate pesticides did result in similar sublethal effects on honeybees to those observed here one could foresee three main consequences. First, if bees have learnt to forage in a certain area and this area becomes contaminated, the bees are likely to persist visiting the contaminated area for longer even if this area becomes less profitable for their foraging. They would therefore be more likely to come into contact with more pesticides and thus may accumulate lethal concentrations. Such consequences are not generally considered when safety protocols are being developed, for example when determining the maximum concentration of a given insecticide that can be safely applied to agricultural crops. 

Second, it is obviously important for the survival of the honeybee colony that the supply of food to the hive be maintained at an optimum. Therefore, it is necessary that foragers are able to learn new food sources when old ones become depleted. Based on these results, if forager bees have been exposed to acetylcholinesterase inhibitors it is extremely likely that they would persist in visiting a depleted food source for longer, inducing sub-optimal foraging. Sub-optimal foraging may have various negative consequences for the hive in terms of nectar and pollen collection. The fertilisation of bee pollination-dependent plants may also be less efficient, which may have large economic and social ramifications on the production of a wide variety of honeybee dependent crops such as almonds, melons, apples, raspberries and lucern (e.g., [36]). Equally important, persisting with a singular particular food source may decrease the variety of food sources visited by the bees, which may represent a significant problem as it has been recently shown that a restricted variety in honeybee diet can be responsible for a decrease in their immune function [37]. 

Third, Ismail *et al.* [34] have shown that treatment of honeybees with the muscarinic agonist pilocarpine improved kin recognition with the consequence of increasing non-kin aggression, whereas the use of the muscarinic antagonist scopolamine decreased kin recognition. One compelling interpretation of these results is that agonistic stimulation of the muscarinic system improved the recall of kin odour, as suggested by the results of Guez *et al.* [15], whereas the antagonistic action of scopolamine was detrimental to the recall of kin odour as suggested by the work of Cano Lozano *et al.* [10]. Although improved kin recognition would not appear to be a problem in natural settings, it may increase overall level of aggression in commercial settings. Commercial beekeepers often place hundred of hives on the same site, and in this condition a drift of population from hive to hive is often observed. However, this “normal” drift of workers could lead to increased overall aggression among hives as a result of improved pesticide-induced kin recognition, potentially leading to detrimental effects. Clearly, more studies evaluating and modeling the sub-lethal effects of these compounds on honeybees are necessary in order to evaluate their possible impacts upon honeybee behaviour.
